# Natural Products Targeting Angiogenesis and Tumor Microenvironment in Gastrointestinal Malignancies

**DOI:** 10.3390/cells15070623

**Published:** 2026-03-31

**Authors:** Idris Arslan

**Affiliations:** Faculty of Science, Molecular Biology and Genetics, Zonguldak Bülent Ecevit University, Zonguldak 67100, Türkiye; idris.arslan@beun.edu.tr

**Keywords:** gastrointestinal, malignancy, angiogenesis, TME, natural products

## Abstract

**Highlights:**

**What are the main findings?**

- Natural compounds modulate angiogenesis and tumor microenvironment in GI cancers
- These effects involve hypoxia, inflammation, and fibroblast-related pathways

**What are the implications of the main findings?**

- Nanoparticle-based delivery enhances the efficacy of natural compounds
- Combination therapies improve anticancer outcomes
- Precision oncology strategies support clinical translation in GI cancers

**Abstract:**

Gastrointestinal malignancies remain among the leading causes of cancer-related morbidity and mortality worldwide, largely due to late diagnosis, aggressive tumor progression, and resistance to conventional therapies. Tumor angiogenesis and the tumor microenvironment (TME) play crucial roles in the initiation, growth, and metastatic dissemination of gastrointestinal cancers. Hypoxia-driven signaling pathways, including hypoxia-inducible factor-1α (HIF-1α), vascular endothelial growth factor (VEGF), transforming growth factor-β (TGF-β), and inflammatory mediators such as NF-κB and MAPK, are key regulators of these processes. Increasing evidence suggests that natural products derived from medicinal plants and other biological sources may modulate these pathways and exhibit anti-angiogenic, anti-inflammatory, and anti-fibrotic properties. This review summarizes recent findings on natural compounds that influence angiogenesis and tumor microenvironment dynamics through the regulation of molecular pathways involved in hypoxia signaling, extracellular matrix remodeling, fibroblast activation, and inflammatory responses. Compounds such as neotuberostemonine, aloperine, silymarin derivatives, tanshinone IIA, berberine, asiatic acid, and phloretin demonstrate promising biological activities in experimental models by targeting pathways including HIF-1α, PI3K/AKT/mTOR, TGF-β/Smad, and NF-κB signaling. However, further studies focusing on gastrointestinal cancer models and clinical validation are required to translate these preclinical observations into effective therapeutic strategies.

## 1. Introduction

Gastrointestinal cancers constitute a diverse group of malignancies affecting various divisions of the gastrointestinal tract. These malignancies can be broadly divided into three groups for classification: hepatobiliary (cancers of the pancreas, liver, gallbladder, and bile ducts), lower gastrointestinal (cancers of the colon, rectum, and anus), and upper gastrointestinal (cancers of the esophagus, stomach, and small intestine). Globally, the incidence of various gastrointestinal cancers varies; pancreatic and colorectal cancers are more prevalent in Europe and North America, whereas esophageal, gastric, and liver cancers are more prevalent in Asia [1].

Together, these illnesses cause 35% of all cancer-related deaths and 26% of all cancer incidence worldwide, making them a major contributor to the global cancer burden [1]. Poor prognosis in the presence of metastatic disease and late-stage identification account for a substantial portion of the morbidity and mortality rates of gastrointestinal malignancies, underscoring the need for additional therapeutic options [2].

Angiogenesis is a common driver in the pathophysiology of all gastrointestinal malignancies, despite the fact that the different types show varying degrees of reliance on this process. When a tumor grows larger than 2 mm, the surrounding tissue can no longer sustain it, resulting in a hypoxic tumor microenvironment (TME) that encourages the development of new blood vessels to carry nutrients and oxygen [3]. Angiogenesis is the most important factor in gastrointestinal malignancies of the stomach, liver, colon, and rectum among those previously discussed [4].

Hepatocellular carcinoma (HCC) is a hypervascular tumor, considering the distinctive vascular supply of the liver. Hepatic artery blood flow accounts for 25% of the total cardiac output during each cardiac cycle [5]. Vascular endothelial growth factor (VEGF), a key signaling molecule in angiogenesis, has been found to be highly expressed in HCC cell lines, tissues, and the blood circulation of patients with HCC, particularly those with aggressive illness [6]. Similarly, individuals with metastatic colorectal cancer (CRC) have much higher VEGF levels, while those with minimal VEGF expression have far higher survival rates [7].

Gastric cancer is generally classified into two major subtypes depending on the Lauren classification, namely, intestinal and diffuse types, both differing in their histological characteristics [8]. The first subtype ordinarily metastasizes to the liver through hematologic spread, whereas the latter subtype depends on peritoneal dissemination. Their angiogenic phenotypes reflect this discrepancy, with the intestinal subtype exhibiting significantly higher VEGF expression as compared to its counterpart [9].

## 2. The Tumor Microenvironment (TME)

Cancers represent complicated ecosystems consisting of tumor cells and a multitude of noncancerous cells, encapsulated in an altered extracellular matrix. The TME includes diverse immune cells, cancer-associated fibroblasts, endothelial cells, pericytes, various additional tissue-resident cells and the extracellular matrix (ECM). Cancer cells trigger significant cellular, molecular, and physicochemical changes within their host tissues. In order to sustain cancer cell survival, local invasion, and metastatic dissemination, a dynamic and reciprocal connection between cancer cells and TME components forms early in tumor progression. The TME regulates a process that encourages angiogenesis to refill the oxygen/nutrient supply and eliminate metabolic waste in order to overcome a hypoxic and acidic microenvironment. Numerous innate and adaptive immune cells infiltrate tumors, which can have both pro- and anti-tumorigenic actions [10].

The TME constitutes a dynamic and heterogeneous milieu that profoundly influences tumorigenesis, progression, and metastatic dissemination, particularly in gastrointestinal malignancies such as colorectal and gastric adenocarcinomas. Pharmacological modulation of the TME using rationally designed drug candidates represents a compelling therapeutic avenue, as these agents can selectively target key stromal components—including cancer-associated fibroblasts, immunosuppressive myeloid populations, and the extracellular matrix—thereby dismantling the supportive niche that facilitates neoplastic growth. By perturbing TME-driven signaling networks, such compounds not only attenuate tumor proliferation but also potentiate the efficacy of conventional cytotoxic or targeted therapies. Importantly, TME-directed therapeutics can impede metastatic cascade events by inhibiting angiogenesis, normalizing aberrant immune surveillance, and restricting matrix remodeling, thereby curtailing invasion and distant organ colonization. Consequently, leveraging TME-targeted interventions offers a dual advantage in gastrointestinal oncology: direct tumoricidal activity and disruption of microenvironmental processes that underpin metastasis.

## 3. Angiogenesis

**Figure 1:**
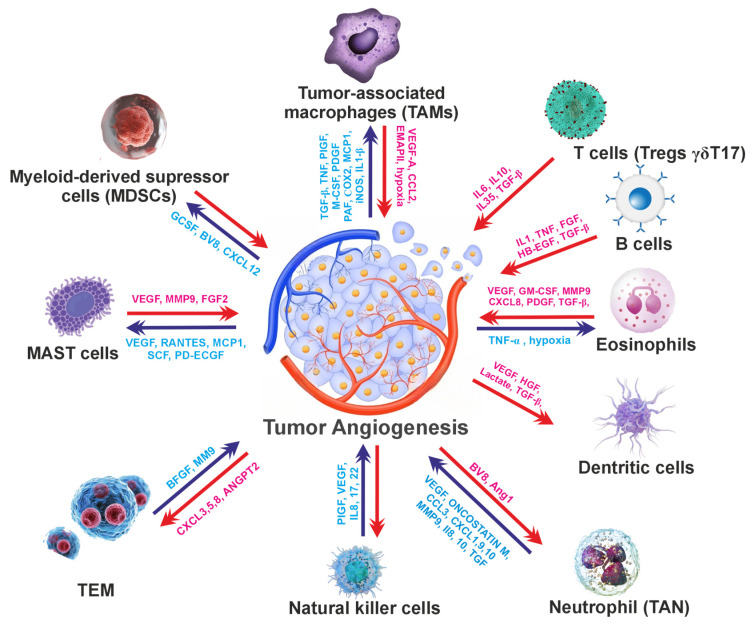
Immune cell-mediated regulation of tumor angiogenesis in the tumor microenvironment. Various immune cells—including tumor-associated macrophages (TAMs), myeloid-derived suppressor cells (MDSCs), mast cells, T cells, B cells, eosinophils, dendritic cells, neutrophils (TANs), natural killer (NK) cells, and tumor-associated endothelial/monocytic cells (TEMs)—contribute to tumor angiogenesis by secreting pro-angiogenic cytokines, chemokines, and growth factors such as VEGF (vascular endothelial growth factor), FGF (fibroblast growth factor), PDGF (platelet-derived growth factor), TGF-β (transforming growth factor), IL-6 (interleukin), IL-8, and MMPs (matrix metalloproteinases). These mediators promote blood vessel formation, tumor growth, and remodeling of the tumor microenvironment.

Angiogenesis is a process in which new blood vessels develop from preexisting capillaries and eventually create a complete, regular, and mature vascular network (Figure 1). This arrangement involves the degradation of the basement membrane and the activation, proliferation, and migration of endothelial cells (ECs), regulated by a range of pro-angiogenic and anti-angiogenic factors [11]. Endothelial cells in healthy adults are almost quiescent, and the frequency of mitosis is only 0.5% under normal physiological conditions [12]. Angiogenesis mostly occurs in embryonic development, tissue repair, the menstrual cycle, muscle growth, and organ lining regeneration through a regular (strictly controlled), scope-limited (occurs locally), and short-lived (days, weeks, or months) mode in the body [13,14]. Angiogenesis mainly contributes to the progression of various malignant tumors, such as melanoma, breast cancer [15], colorectal cancer [16], and gastrointestinal cancers [17,18,19].

When tumor cell growth outpaces the capacity of existing vasculature, regions of low oxygen develop and, in response, tumors start producing factors that promote unorganized and immoderate blood vessel formation [20]. This process is mostly regulated by a group of proteins called hypoxia-inducible factors (HIFs), which become stabilized under low oxygen conditions and activate genes participating in blood vessel growth, such as VEGF, fibroblast growth factor (FGF), and platelet-derived growth factor (PDGF) [21]. Overexpression of HIF-1α is commonly seen in gastrointestinal malignancies and is linked to worse clinical outcomes as well as poor response to therapy [22,23].

Oncostatin M was first identified as a growth regulator of tumor cells [24]. Numerous studies were thus focused on cancer research and reported that the levels of oncostatin M were upregulated in various patients with cancer as well as tumor tissues [25,26,27]. However, recent studies revealed that oncostatin M is a pro-inflammatory factor and is released in a variety of cells, including dendritic cells, stimulated T cells, macrophages, monocytes, and neutrophils [28].

Angiopoietin-2 (Ang2) is a member of the Ang family, which plays an important role in angiogenesis during the development and growth of human cancers. Ang2’s role in angiogenesis is generally considered as an antagonist for Ang1, inhibiting Ang1-promoted Tie2 signaling, which is critical for blood vessel maturation and stabilization [29].

**Figure 2:**
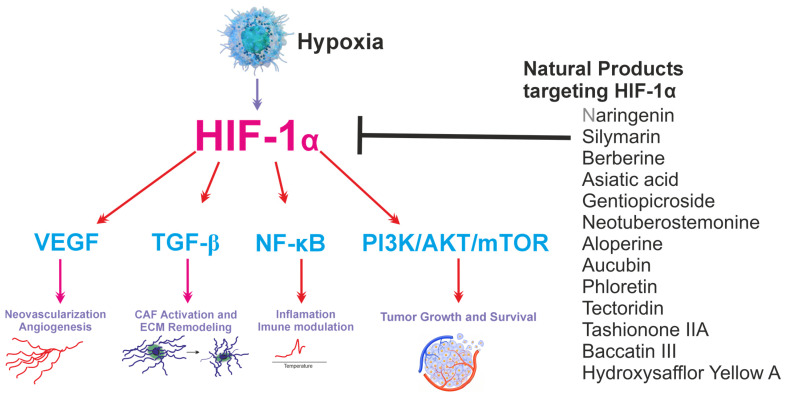
Schematic representation of natural compounds targeting angiogenesis and tumor microenvironment-related signaling pathways in gastrointestinal malignancies. Through the regulation of these pathways, natural compounds suppress pro-angiogenic signaling, inflammatory responses, fibroblast activation, and extracellular matrix remodeling within the tumor microenvironment. Hypoxia-inducible factor-1α (HIF-1α); vascular endothelial growth factor (VEGF); cancer-associated fibroblast (CAF); transforming growth factor-beta (TGF-β); nuclear factor kappa B (NF-κB); phosphatidylinositol 3-kinase (PI3K); protein kinase B (Akt); mammalian target of rapamycin (mTOR).

Several natural compounds have been reported to interfere with hypoxia-related signaling pathways, particularly the HIF-1α axis, which plays a central role in tumor angiogenesis and adaptation to hypoxic tumor microenvironments. Compounds such as neotuberostemonine and aloperine have been shown to suppress HIF-1α activity and downstream mediators, including TGF-β, FGF2, and PI3K/AKT/mTOR signaling, thereby reducing fibroblast activation, inflammatory signaling, and extracellular matrix remodeling in tumor microenvironments as shown in Figure 2. Although most evidence comes from pulmonary fibrosis or other non-gastrointestinal models, these hypoxia-responsive pathways are broadly conserved across tissues, suggesting potential relevance for gastrointestinal cancers. Nevertheless, the absence of gastrointestinal-specific in vitro, in vivo, or clinical studies represents a key limitation and highlights the need for targeted investigations in GI cancer systems.

Natural compounds represent compounds that have been isolated from higher plants, fungi, and animals. Along with their structural analogues, they have had and continue to have a relevant role in anticancer drug discovery, by offering the advantage of a stable chemical structure with higher specificity for target proteins. The aim of this review is to demonstrate the potential of natural products that directly target factors regulating gastrointestinal inflammation in preclinical studies. The major natural compounds reported to modulate angiogenesis and tumor microenvironment-related pathways are summarized in Table 1.

Neotuberostemonine, a natural alkaloid isolated from *Stemona tuberosa* (Stemonaceae), suppresses the activation of hypoxia-exposed lung fibroblasts. A recent study showed that PLFs (primary mouse lung fibroblasts) were activated and differentiated after exposure to 1% O_2_ or treatment with CoCl_2_ (100 μmol/L), evidenced by markedly increased protein or mRNA expression of HIF-1α, TGF-β, FGF2, α-SMA and Col-1α/3α, which was inhibited after silencing HIF-1α, suggesting that the activation of fibroblasts was HIF-1α-dependent. The results demonstrated that neotuberostemonine (e.g., 10–50 μM) significantly suppressed hypoxia-induced activation of primary mouse lung fibroblasts (PLFs) exposed to 1% O_2_ or CoCl_2_ (100 μM) by inhibiting HIF-1α signaling, while the inhibitory action of neotuberostemonine was abrogated by co-treatment with MG132, a proteasome inhibitor. Taken together, these results demonstrate that neotuberostemonine blocks the protein expression of HIF-1α and its downstream factors TGF-β, FGF2 and α-SMA both in hypoxia-exposed fibroblasts and in lung tissues of BLM-treated mice [30].


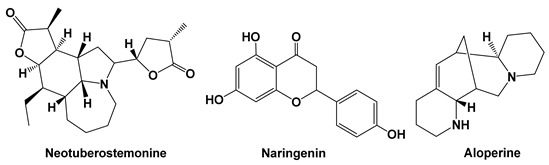


Naringenin is a flavonoid belonging to the flavanone subclass. It is widely distributed in several *Citrus* fruits, bergamot, tomatoes and other fruits, and is also found in its glycoside form. Naringenin (10–100 μM) inhibited oncostatin M release in neutrophil-like differentiated dHL-60 cells via suppression of the PI3K/Akt/NF-κB pathway [31].

Clinical research on naringenin remains limited and has primarily focused on safety and pharmacokinetics rather than definitive therapeutic efficacy. In the most well-characterized human study, 18 healthy adults participated in a randomized, controlled, single ascending dose clinical trial in which they received 150 mg, 300 mg, 600 mg, or 900 mg of naringenin from a citrus extract or placebo. This study reported no significant adverse events across doses and demonstrated dose-proportional increases in serum naringenin concentrations over 24 h, establishing an initial safety and pharmacokinetic profile in humans [32].

Aloperine is a quinolizidine alkaloid extracted from *Sophora alopecuroides* L., primarily used as a free base (pure) in its natural form. A recent study showed that aloperine (10–40 μM) inhibited the proliferation of PDGF-BB-stimulated mouse lung fibroblasts and suppressed PI3K/AKT/mTOR and TGF-β/Smad signaling pathways [33].

The results revealed that aloperine might be ascribed to inhibition of the PI3K/AKT/mTOR and TGF-β/Smad signaling pathways.

Silymarin, the major constituent of milk thistle (*Silybum marianum*) extract and a mixture of some flavonolignans, significantly reduces serum levels of IL-1 alpha, IL-8, C3 and C4 after 8 weeks compared to pre-treatment levels. Meloxicam significantly elevates serum levels of IL-1 alpha, while IL-8 does not significantly change compared to the pre-treatment value [51]. Silibinin is the most active natural compound of silymarin and has been reported to downregulate MMP2 expression via Jak2/STAT3 pathway and inhibit the migration and invasive potential in MDA-MB-231 cells [34].

Importantly, silibinin has also been evaluated in clinical settings. A phase I clinical trial in prostate cancer patients demonstrated that oral silybin-phytosome was well tolerated and established a recommended phase II dose [35].

Flaig and colleagues (2007) [35] designed a study to assess the toxicity of high-dose silybin-phytosome and recommend a phase II dose. Silybin-phytosome was administered orally to prostate cancer patients, giving 2.5–20 g daily, in three divided doses. Each course was 4 weeks in duration. Thirteen patients received a total of 91 courses of silybin-phytosome. Baseline patient characteristics included: median age of 70 years, median baseline prostate-specific antigen (PSA) of 4.3 ng/mL, and a median ECOG performance status of 0. The most prominent adverse event was hyperbilirubinemia, with grade 1–2 bilirubin elevations in nine of the 13 patients. The only grade 3 toxicity observed was elevation of alanine aminotransferase (ALT) in one patient; no grade 4 toxicity was noted. No objective PSA responses were observed. We conclude that 13 g of oral silybin-phytosome daily, in three divided doses, appears to be well tolerated in patients with advanced prostate cancer and is the recommended phase II dose. Asymptomatic liver toxicity is the most commonly seen adverse event [35].


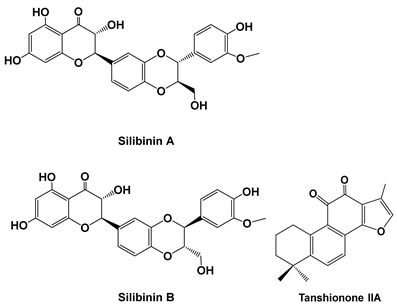


Tanshinone IIA is a lipophilic diterpene quinone extracted from the Chinese herb *Salvia miltiorrhiza* Bunge. Studies showed that Tanshinone IIA (10–30 mg/kg, in vivo bleomycin-induced pulmonary fibrosis rat model) suppressed BLM-induced increased expression of TNF-α, IL-1β, IL-6, COX-2, PGE2, malondialdehyde, iNOS and NOS [36].

Baccatin III (BAC) is the precursor of the semisynthesis of paclitaxel, a well-known anticancer drug from the Pacific yew tree. Nie et al. (2019) [37] demonstrated that Baccatin III (5–20 mg/kg, in vivo; 10–50 μM, in vitro fibroblast models) reduced inflammatory infiltration, secretion of the pro-fibrotic mediator TGF-β1, and extracellular matrix deposition in a dose-dependent manner. Moreover, it suppressed the β1 signaling pathway and Smad2/3/TGF [37].


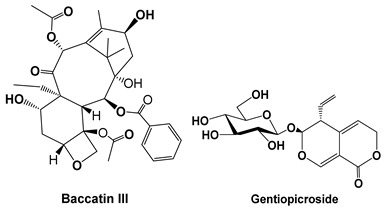


Gentiopicroside is a natural secoiridoid glycoside from gentian species of medicinal plants, such as *Gentiana scabra*, *Gentiana lutea*, *Swertia pseudochinensis* and *Swertia mussotii*, which are commonly used as medicinal herbs in China and Europe [52,53]. Gentiopicroside (50–200 mg/kg, in vivo bleomycin-induced pulmonary fibrosis mouse model; 10–100 μM, in vitro A549 cells) remarkably ameliorated inflammatory and fibrotic responses in the lungs of pulmonary fibrosis mice, as confirmed by histopathological examinations, including light microscopy and transmission electron microscopy. Furthermore, gentiopicroside significantly decreased the levels of inflammatory cytokines such as TNF-α and IL-1β in bronchoalveolar lavage fluid and reduced the content of hydroxyproline in the lungs. Similarly, gentiopicroside significantly downregulated the expression of TGF-β1 and connective tissue growth factor (CTGF) in the lungs of pulmonary fibrosis mice. Also, gentiopicroside suppressed in vitro epithelial–mesenchymal transition of A549 cells stimulated by TGF-β1 in a dose-dependent manner. These findings suggest that alveolar epithelial cells and TGF-β1 may be the main target cells and molecule of gentiopicroside [38].

Astilbin was reported to ameliorate pulmonary fibrosis via blockade of the Hedgehog signaling pathway through a reduction in pathological scores and collagen deposition, with a decrease in α-SMA and Snail and an increase in E-cadherin and SP-C (surfactan protein-C) (20–80 mg/kg in vivo; 10–50 μM in vitro AEC-II and L929 cells). The expression of mesenchymal markers and the loss of epithelial cell markers were assayed to determine the anti-fibrotic activity of astilbin on TGF-β1-treated AEC-II and L929 cells and bleomycin-treated mice [39].


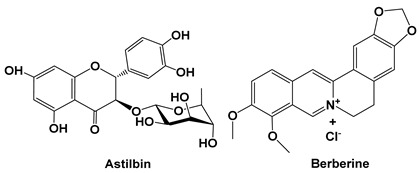


Astilbin is a dihydroflavonol rhamnoside and a significant natural compound in the treatment of immunologically related diseases. Several studies have reported that astilbin ameliorates experimental autoimmune myasthenia gravis and imiquimod-induced psoriasis-like skin lesions [54,55]. However, astilbin has also been reported to ameliorate cisplatin-induced nephrotoxicity by reducing oxidative stress and inflammation [56]. On the other hand, astilbin significantly inhibited the 3-hydroxy-3-methylglutaryl coenzyme A reductase (HMG-CoA) enzyme on Vero cells [57].

Berberine is a plant quaternary ammonium salt from the group of isoquinoline alkaloids (2,3-methylenedioxy-9, 10-dimethoxyprotoberberine chloride; C_20_H_18_NO_4_^+^) that is derived from the Chinese medicinal plant Coptis chinensis. It has been identified to have multiple pharmacological activities, including regulating glucose and cholesterol levels, anti-obesity effects and anti-diabetic effects [58].

Orally administered berberine (50–200 mg/kg, in vivo mouse model; 10–50 μM, in vitro fibroblast cells) ameliorates bleomycin-induced pulmonary fibrosis in mice by promoting activation of PPAR-γ and subsequent expression of HGF in the colon [40]. Oral or rectal administration of berberine displayed marked alleviation of bleomycin-induced pulmonary fibrosis in a mouse model. However, the anti-pulmonary fibrosis activity of berberine disappeared when given by an intravenous injection, implying that it functioned in a gut-dependent manner. Additionally, berberine induced both mRNA and protein levels of HGF and PTEN in the colon. Moreover, SU11274, a selective c-Met/HGFR inhibitor, but not BPV, a PTEN inhibitor, abolished the anti-pulmonary fibrosis effect of berberine. On the other hand, berberine preferentially triggered in vitro expression of HGF in fibroblast cells more than epithelial, preadipocyte and endothelial cells. Berberine is able to enter into the protoplasm, activate PPAR-γ and work synergistically with 15-deoxy-Δ12, 14-prostaglandin J2, as shown by an upregulation of CD36 and adipocyte fatty acid-binding protein 2 mRNA expression, nuclear translocation and DNA-binding activity of PPAR-γ both in vitro and in vivo [40].

Importantly, berberine has also been evaluated in clinical trials for preventing colorectal adenoma recurrence and neoplasm occurrence. Berberine has been reported as a safe and effective pharmacological agent that reduces colorectal adenoma recurrence after polypectomy. In a retrospective cohort study that was an extended follow-up of a previous clinical trial during the post-treatment observational phase, Tan and colleagues (2025) [41] aimed to evaluate the long-term protective effects of berberine on adenoma recurrence. Among 895 patients who finished the previous 2-year randomized trial, they recruited 781 patients at seven clinical centers across six provinces in China. The primary outcome was adenoma recurrence. Between 29 December 2018 and 10 October 2024, 648 patients underwent at least one colonoscopy during the follow-up. The protective effects of berberine persist for at least 6 years after treatment cessation, with a lower adenoma recurrence rate (34.7% vs. 52.1%) and a lower neoplasm occurrence rate (63.4% vs. 71.0%). The results revealed that berberine may serve as a potential long-term preventive agent against adenoma recurrence after polypectomy [41].

Asiatic acid was reported to suppress TGF-β1-induced expression of collagen type I, inhibit Smad 2/3 phosphorylation and plasminogen activator inhibitor-1 (PAI-1) expression, and elevate Smad 7 protein levels. Additionally, the effects of asiatic acid on keloid fibroblasts could be abrogated by PPAR-γ antagonist GW9662 and by silencing PPAR-γ [42].


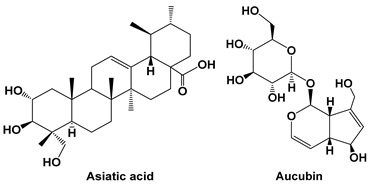


Lv and colleagues (2017) [43] showed that asiatic acid treatment (10–40 μM, in vitro; 25–100 mg/kg, in vivo models) effectively protected against LPS/d-GalN-induced fulminant hepatic failure by lessening lethality; decreasing alanine transaminase and aspartate aminotransferase levels, IL-1β, IL-6, and tumor necrosis factor-α production, malondialdehyde formation, myeloperoxidase level and reactive oxygen species generation (i.e., H_2_O_2_, NO, and O_2_^−^); and increasing glutathione and superoxide dismutase contents. Furthermore, asiatic acid administration significantly inhibited MAPK and NF-κB signaling pathway activation via the partial induction of programmed cell death 4 (PDCD4) protein expression [43].

A phase 1 clinical trial (NCT05591027) was conducted to evaluate the safety and tolerability of *Centella asiatica* extract in humans. This phase I study was a randomized, double-blind, placebo-controlled, clinical trial of 48 participants to evaluate safety, tolerability, and biological signatures of the target engagement of brain neuronal viability, oxidative stress, and brain mitochondrial activity of a Centella asiatica water extract product (CAP) in older adults aged 60–85 years with mild cognitive impairment or mild Alzheimer’s disease (AD) [44].

Aucubin is a natural constituent with a monoterpene cyclic ring system, found in a wide range of higher plants such as *Aucuba japonica* and *Plantago asiatica* [59]. Aucubin was reported to reduce the intra-pulmonary collagen disposition and inflammatory injury induced by bleomycin. Similarly, aucubin reduced the expression of pro-fibrotic protein transforming growth factor (TGF)-β1 and α-smooth muscle actin (α-SMA) in pulmonary fibrosis mice (20–80 mg/kg, in vivo mouse model; 10–50 μM, in vitro fibroblasts). Moreover, aucubin inhibited the mRNA and protein expression of Ki67 and proliferating cell nuclear antigen (PCNA) induced by TGF-β1 and reduced the cell proliferation in murine fibroblast cells, NIH3T3. It also reduced the collagen synthesis and αSMA expression induced by TGF-β1 in fibroblasts [45].


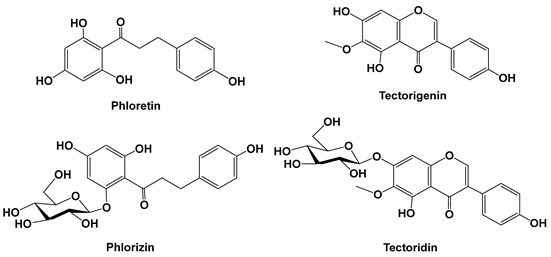


Phloretin is a naturally occurring dihydrochalcone that belongs to the chalcone class of flavonoids. Phloretin was reported to inhibit prostaglandin E_2_, decrease COX-2 expression, and suppress IL-8, monocyte chemotactic protein 1, and IL-6 production. Furthermore, it also decreased ICAM-1 gene and protein expression and suppressed monocyte adhesion to inflammatory cells (10–100 μM, in vitro A549 cells). Phloretin also significantly blocked Akt and MAPK phosphorylation and decreased NF-κB subunit p65 protein translocation into the nucleus. Moreover, ICAM-1 and COX-2 expression was suppressed by pre-treatment with both MAPK inhibitors and phloretin in inflammatory A549 cells. However, phlorizin, a derivative of phloretin, did not suppress the inflammatory response in IL-1β-stimulated A549 cells. These results suggest that phloretin might have an anti-inflammatory effect by inhibiting pro-inflammatory cytokine, COX-2, and ICAM-1 expression via blocking the NF-κB and MAPK signaling pathways [46]. Phlorizin is the glycosylated derivative (glycoside) of phloretin, which acts as a strong, non-selective SGLT1/SGLT2 inhibitor to block glucose absorption [60].

Tectorigenin and tectoridin are the major constituents of the rhizomes of the iridaceous plant *Belamcanda chinensis*, well known as a Chinese traditional medicine for the treatment of inflammatory diseases. Tectorigenin and tectoridin were reported to show anti-inflammatory activity towards Raw 264.7 cells activated with IFN-γ/LPS and pre-treated with tectorigenin. Tectorigenin inhibited (10–50 μM, in vitro RAW 264.7 macrophages) the expression of inducible nitric oxide synthase (iNOS), the production of nitric oxide (NO) and the secretion of IL-1β dose-dependently. It also decreased the expression of cyclooxigenase (COX)-2 and the production of prostaglandin E_2_ (PGE_2_) in a dose-dependent manner [47].

Tectoridin was reported as a PGE2 inhibitor due to the inhibition of COX-2 induction in rat peritoneal macrophages stimulated by either protein kinase C activator (12-o-tetradecanoyphorbol 13-acetate: TPA) or the endomembrane Ca^2+^-ATPase inhibitor (thapsigargin) [48].


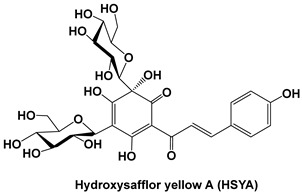


Hydroxysafflor yellow A (HSYA) (6-hydroxykaempferol 3,6-di-*O*-*β*-glucoside-7-*O*-*β*-glucuronide) is an active ingredient of the traditional Chinese herbal medicine *Carthamus tinctorius* [49]. A recent study showed that treatment with HSYA ameliorated lung inflammation, increased oxygen partial pressure (PaO_2_) (HSYA 80.0 mg·kg^−1^) and decreased CO_2_ partial pressure (PaCO_2_) (HSYA 53.3, 80.0 mg·kg^−1^) in lung inflammation and pulmonary fibrosis induced by bleomycin, 53.3–80 mg/kg, in an in vivo rat model. Furthermore, the mRNA expression of TNF-α, IL-1β, and IL-6 and the number of NF-κB p65-positive cells were lower in HSYA 53.3 and 80.0 mg·kg^−1^ groups than those in the model group [50].

In addition to their biological activities, consideration of the origin and pharmaceutical forms of the natural compounds discussed in this review is essential. Most of these bioactive molecules are primarily isolated from higher plants, including *Stemona tuberosa* (neotuberostemonine), *Sophora alopecuroides* (aloperine), *Silybum marianum* (silymarin/silibinin), *Salvia miltiorrhiza* (tanshinone IIA), *Coptis chinensis* (berberine), and *Centella asiatica* (asiatic acid). In terms of availability, many of these compounds are commercially marketed as standardized plant extracts or purified natural products, particularly as dietary supplements or herbal formulations. However, certain compounds—especially those with well-established pharmacological relevance—are also produced as semi-synthetic or fully synthetic derivatives to enhance their bioavailability, stability, and pharmacokinetic profiles. Therefore, both naturally derived and synthetic forms of these compounds may be encountered in experimental and clinical settings, which should be taken into account when interpreting their therapeutic potential. This distinction may have important implications for their pharmacological application and clinical translation, as discussed in the following section.

## 4. Combination Strategies of Natural Compounds with Conventional Chemotherapeutics in Gastrointestinal Malignancies

The combination of natural bioactive compounds with conventional chemotherapeutic agents has emerged as a promising strategy to enhance therapeutic efficacy and overcome drug resistance in gastrointestinal malignancies. Standard treatments such as 5-fluorouracil (5-FU) and oxaliplatin remain the backbone of chemotherapy for colorectal and gastric cancers; however, their clinical utility is often limited by systemic toxicity, intrinsic or acquired resistance, and tumor microenvironment-mediated protective mechanisms. In this context, phytochemicals with multi-targeted biological activities may serve as effective adjuvants that sensitize tumor cells to chemotherapy while simultaneously modulating the TME [61].

A growing body of preclinical evidence indicates that several natural compounds discussed in this review can potentiate the antitumor activity of conventional drugs. For instance, berberine has been shown to enhance the cytotoxic effects of 5-FU in colorectal cancer models by modulating key survival pathways, such as PI3K/AKT, and by inducing apoptosis through mitochondrial-dependent mechanisms. In addition, berberine has been reported to suppress NF-κB signaling, thereby reducing inflammation-associated chemoresistance and attenuating pro-survival signaling within the TME [62].

Similarly, silibinin, the major active constituent of silymarin, has demonstrated the ability to increase chemosensitivity to both 5-FU and oxaliplatin. Mechanistically, silibinin inhibits the STAT3 and NF-κB signaling pathways, leading to reduced tumor cell proliferation, decreased angiogenic signaling, and impaired metastatic potential. Importantly, silibinin-mediated downregulation of MMPs may further limit extracellular matrix remodeling and tumor invasion, thereby complementing the cytotoxic effects of chemotherapy [63,64]. 

**Figure 3:**
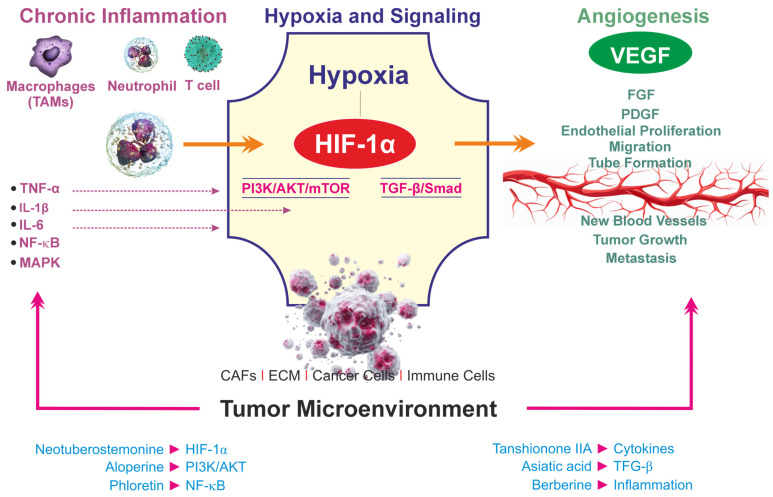
A schematic view of the interconnection between chronic inflammation and angiogenesis in the TME. Various factors, such as CAFs (cancer-associated fibroblasts), TGF-β (transforming growth factor), PDGF (platelet-derived growth factor), FGF (fibroblast growth factor), IL-6 and IL-1β (interleukin), NF-κB (nuclear factor kappa B), TNF (tissue necrosis factor), and phosphatidylinositol 3-kinase (PI3K), regulate chronic inflammation and the TME.

Flavonoids such as naringenin and phloretin have also been reported to exert synergistic effects with chemotherapeutic agents [65,66]. Naringenin enhances drug-induced apoptosis and reduces oxidative stress-mediated resistance by modulating the PI3K/AKT and MAPK signaling pathways. Phloretin, through inhibition of NF-κB and Akt signaling, contributes to the suppression of inflammatory cytokine production and may sensitize tumor cells to chemotherapeutic stress [65,66]. These effects are particularly relevant in gastrointestinal cancers, where chronic inflammation plays a central role in tumor progression and therapy resistance as shown in Figure 3.

Another important aspect of combination therapy is the modulation of hypoxia-related signaling pathways. Natural compounds such as neotuberostemonine and aloperine, which inhibit HIF-1α activity, may improve the response to chemotherapy by reducing hypoxia-driven drug resistance. Since hypoxic regions within tumors are often less responsive to cytotoxic agents, targeting HIF-1α and its downstream mediators (e.g., VEGF, TGF-β) may enhance drug delivery and efficacy by normalizing tumor vasculature and reducing angiogenesis [30]. 

Furthermore, compounds such as asiatic acid and gentiopicroside, which regulate TGF-β/Smad signaling, may counteract chemotherapy-induced epithelial–mesenchymal transition (EMT) and fibrosis, both of which are associated with drug resistance and metastatic progression. By attenuating stromal activation and fibroblast-mediated protective niches, these natural agents may disrupt the supportive microenvironment that enables tumor survival during chemotherapy [67].

Importantly, recent experimental studies have demonstrated that natural compounds can directly potentiate oxaliplatin activity. For example, polydatin has been shown to enhance oxaliplatin-induced cytotoxicity in colon cancer cells by promoting reactive oxygen species (ROS) accumulation, DNA damage, and endoplasmic reticulum stress-mediated apoptosis [68]. These findings support the concept that phytochemicals can act as chemosensitizers through multiple converging molecular mechanisms.

In addition to enhancing efficacy, combination strategies may also allow dose reduction in chemotherapeutic agents, thereby minimizing adverse effects while maintaining therapeutic benefit. This is particularly relevant for oxaliplatin-based regimens, where cumulative neurotoxicity remains a major clinical limitation [69]. Despite these promising findings, most available evidence is still derived from preclinical models. Therefore, well-designed gastrointestinal cancer-specific studies and clinical trials are required to validate these combinatorial approaches and to determine optimal dosing strategies, pharmacokinetic interactions, and safety profiles.

Overall, the integration of natural compounds with conventional chemotherapeutics represents a rational and multifaceted therapeutic strategy. By simultaneously targeting tumor cells and the tumor microenvironment—including angiogenesis, inflammation, hypoxia signaling, and stromal interactions—these combination approaches hold significant potential to improve treatment outcomes in gastrointestinal malignancies.

## 5. Discussion

Angiogenesis and tumor microenvironment (TME) remodeling represent central hallmarks of gastrointestinal tumor progression. The dynamic interaction between tumor cells and stromal components—including immune cells, cancer-associated fibroblasts, endothelial cells, and extracellular matrix elements—creates a complex microenvironment that promotes tumor growth, invasion, and metastatic dissemination. Hypoxia is a critical driver of these processes, primarily through the activation of hypoxia-responsive signaling pathways such as HIF-1α, VEGF, TGF-β, NF-κB, and MAPK. These pathways collectively regulate endothelial cell proliferation, inflammatory signaling, fibroblast activation, and extracellular matrix remodeling, thereby establishing a pro-angiogenic and tumor-supportive microenvironment.

Importantly, emerging evidence supports the therapeutic potential of combining natural compounds with conventional chemotherapeutic agents in gastrointestinal malignancies. Combinatorial strategies may enhance chemosensitivity to agents such as 5-fluorouracil and oxaliplatin by targeting multiple oncogenic pathways, including PI3K/AKT, NF-κB, and hypoxia-related signaling. These findings further highlight the relevance of phytochemicals as adjuvant agents capable of modulating both tumor cells and the tumor microenvironment, thereby improving overall treatment efficacy.

Many mechanistic studies have utilized non-oncological models, particularly pulmonary fibrosis systems, which may not fully recapitulate the complexity of gastrointestinal tumors. Although these models provide valuable insights into hypoxia-responsive and fibrotic pathways, their direct translational relevance to gastrointestinal oncology remains uncertain. Several natural compounds reviewed here have reached early clinical studies, offering preliminary data on safety and pharmacokinetics; for example, naringenin is well tolerated in humans with predictable oral pharmacokinetics. Nonetheless, gastrointestinal-specific mechanistic and clinical investigations are essential to validate these findings and to guide future translational efforts in GI cancer research.

Importantly, several natural compounds discussed in this review have already been evaluated in early clinical studies, providing preliminary insights into their safety and pharmacokinetic profiles. For example, clinical investigations have demonstrated that naringenin is well tolerated in humans and exhibits predictable pharmacokinetics following oral administration. Similarly, silybin-phytosome has been evaluated in a phase I clinical trial in prostate cancer patients, where high-dose administration was generally well tolerated. In addition, long-term follow-up data from clinical trials investigating berberine have demonstrated its potential role in reducing colorectal adenoma recurrence after polypectomy. These findings highlight the translational potential of natural products and emphasize the need for further clinical evaluation in gastrointestinal malignancies.

Future research should therefore focus on several key areas. First, mechanistic studies using gastrointestinal tumor-specific cellular and animal models are needed to better define the anti-angiogenic and microenvironment-modulating properties of these compounds. Second, the integration of natural compounds with existing therapeutic strategies—including chemotherapy, targeted therapies, and immunotherapy—may provide synergistic effects and improve treatment outcomes. Third, advances in drug delivery technologies, such as nanoparticle-based formulations and targeted drug delivery systems, may overcome limitations related to poor bioavailability and pharmacokinetic instability that often affect plant-derived compounds.

Finally, well-designed randomized clinical trials will be essential to determine whether the promising biological activities observed in experimental studies can be translated into clinically meaningful benefits for patients with gastrointestinal malignancies. Integrating natural product research with translational oncology approaches may ultimately lead to the development of novel multi-targeted therapeutic strategies that simultaneously disrupt tumor angiogenesis and microenvironmental support systems.

In addition, the application of precision oncology approaches could enable patient-specific selection of natural compounds and combination therapies, maximizing therapeutic efficacy while minimizing off-target effects. Coupling these strategies with nanoparticle-based delivery systems may further enhance compound bioavailability and tumor-targeted activity, accelerating their clinical translation in gastrointestinal cancers.

## 6. Conclusions

Natural products represent a rich and biologically diverse source of compounds capable of modulating key signaling pathways involved in angiogenesis and tumor microenvironment regulation. The evidence summarized in this review demonstrates that multiple natural compounds can influence the central molecular pathways, inflammatory responses, and stromal activation that contribute to gastrointestinal tumor progression.

Although the majority of available studies remain limited to preclinical experimental models, the growing number of early clinical investigations evaluating compounds such as naringenin, silybin derivatives, and berberine provides encouraging evidence for their safety and translational potential. In particular, clinical studies demonstrating reduced colorectal adenoma recurrence following berberine treatment highlight the possibility that natural products may contribute not only to therapeutic interventions but also to chemoprevention strategies in gastrointestinal cancers.

Nevertheless, substantial challenges remain before these compounds can be integrated into routine clinical practice. Future studies should prioritize gastrointestinal cancer-specific experimental models, comprehensive pharmacokinetic analyses, and well-designed randomized clinical trials to establish their efficacy and safety profiles. In addition, combination approaches integrating natural compounds with current anti-angiogenic therapies, immunotherapy, or targeted agents may represent promising strategies to enhance therapeutic outcomes.

Overall, continued interdisciplinary research combining natural product pharmacology, tumor microenvironment biology, and clinical oncology will be essential to fully explore the therapeutic potential of natural compounds. Such efforts may ultimately facilitate the development of novel multi-targeted treatment strategies aimed at simultaneously disrupting angiogenesis and tumor-supportive microenvironmental processes in gastrointestinal malignancies.

Future research should prioritize gastrointestinal-specific investigations to validate the mechanistic effects of natural compounds on tumor microenvironments. Specifically, in vitro studies using GI cancer cell lines and patient-derived organoids, as well as in vivo studies employing gastrointestinal PDX models, are needed. Translational studies should focus on pharmacokinetics, bioavailability, and combinatorial strategies with existing chemotherapeutics to assess efficacy and safety in GI contexts. Early-phase clinical trials targeting gastrointestinal cancers are warranted for promising candidates like naringenin and neotuberostemonine, with endpoints including tumor progression, angiogenesis, and immune modulation. Additionally, prioritization of molecular targets based on pathway specificity and preclinical efficacy will guide the rational selection of compounds for clinical development.

## Figures and Tables

**Table 1:** Natural compounds, their chemical classes, and their targeting of the TME and angiogenesis.

Compounds	Compound Class	Major Molecular Targets/Pathways	Anti-Angiogenic Mechanisms/ Tumor Microenvironment (TME) Effects	Evidence Level	Reference
Neotuberostemonine	Alkaloid (stemona alkaloid)	HIF-1α, TGF-β, FGF2 signaling	Suppresses hypoxia-induced HIF-1α activity and downstream pro-angiogenic mediators/ Inhibits fibroblast activation and extracellular matrix remodeling	Preclinical	[30]
Naringenin	Flavonoid	PI3K/Akt/NF-κB	Suppresses angiogenesis via inhibition of inflammatory signaling/ Reduces cytokine release and inflammatory signaling in tumor-associated immune cells	Preclinical + human study/Phase I safety and pharmacokinetics study	[31,32]
Aloperine	Alkaloid (quinolizidine alkaloid)	PI3K/AKT/mTOR, TGF-β/Smad	Inhibits signaling pathways associated with angiogenic and fibrotic responses/ Suppresses fibroblast proliferation and myofibroblast differentiation	Preclinical	[33]
Silymarin/Silibinin	Flavonolignans	JAK2/STAT3, MMP-2	Reduces tumor cell invasion and angiogenesis-associated matrix degradation/ Inhibits ECM remodeling and tumor cell migration	Preclinical + clinical trial	[34,35]
Tanshinone IIA	Diterpenoid (diterpene quinone)	TNF-α, IL-1β, COX-2, iNOS	Suppresses inflammatory mediators that contribute to angiogenesis/ Reduces inflammatory cytokine production in TME	Preclinical	[36]
Baccatin III	Diterpenoid (taxane-type terpenoid)	TGF-β1/Smad signaling	Inhibits pro-fibrotic signaling pathways associated with angiogenic microenvironment formation/ Reduces fibroblast activation and collagen deposition	Preclinical	[37]
Gentiopicroside	Iridoid glycoside (secoiridoid)	TGF-β1, CTGF	Modulates fibrotic signaling pathways indirectly associated with angiogenesis/ Suppresses epithelial–mesenchymal transition (EMT) and inflammatory responses	Preclinical	[38]
Astilbin	Flavonoid	Hedgehog signaling	Indirect suppression of angiogenesis via inhibition of fibrotic signaling/ Reduces EMT and stromal activation in the microenvironment	Preclinical	[39]
Berberine	Alkaloid (isoquinoline alkaloid)	PPAR-γ, HGF signaling	Modulates angiogenesis-associated inflammatory and metabolic signaling/ Alters gut-derived signaling and stromal activation	Preclinical + clinical studies	[40,41]
Asiatic acid	Triterpenoid	TGF-β/Smad, PPAR-γ	Inhibits pro-angiogenic fibrotic signaling/ Reduces inflammatory cytokines and fibroblast activation	Preclinical + phase I study	[42,43,44]
Aucubin	Iridoid glycoside	TGF-β1, α-SMA	Suppresses fibroblast-mediated angiogenic stromal activation/ Reduces collagen synthesis and fibroblast proliferation	Preclinical	[45]
Phloretin	Flavonoid (chalcone)	NF-κB, MAPK, Akt	Decreases angiogenesis-related inflammatory signaling/ Suppresses cytokine, COX-2, and ICAM-1 expression	Preclinical	[46]
Tectorigenin	Isoflavonoids	COX-2, iNOS, PGE2	Inhibits inflammatory mediators involved in angiogenic signaling/ Reduces macrophage-mediated inflammatory responses	Preclinical	[47,48]
HSYA	Flavonoid (quinochalcone)	NF-κB, TNF-α, IL-1β	Suppresses inflammation-driven angiogenesis/ Reduces inflammatory cytokine production in lung injury models	Preclinical	[49,50]

## Data Availability

No new data were created or analyzed in this study.

## Abbreviations

The following abbreviations are used in this manuscript:

TME	Tumor microenvironment
HCC	Hepatocellular carcinoma
VEGF	Vascular endothelial growth factor
TGF-β	Transforming growth factor-beta
MCC	Metastatic colorectal cancer
HIF-1α	Hypoxia-inducible factor-1alpha
ECM	Extracellular matrix
HIFs	Hypoxia-inducible factors
Ang1	Angiopoietin-1
Ang2	Angiopoietin-2
ECs	Endothelial cells
FGF	Fibroblast growth factor
Akt	Protein kinase B
PI3K	Phosphatidylinositol 3-kinase
mTOR	Mammalian target of rapamycin
IL	Interleukin
PLFs	Primary mouse lung fibroblasts
PaO_2_	Oxygen partial pressure
HSYA	Hydroxysafflor yellow A
COX	Cyclooxigenase
NF-κB	Nuclear factor kappa B
CTGF	Connective tissue growth factor
PSA	Prostate-specific antigen
HMG-CoA	3-hydroxy-3-methylglutaryl coenzyme A reductase
PPAR-γ	Peroxisome proliferator-activated receptor gamma
PDGF	Platelet-derived growth factor
MMPs	Matrix metalloproteinases
CAFs	Cancer-associated fibroblasts
